# Incidence, Timing, and Causes of Late Bleeding After TAVR in an Asian Cohort

**DOI:** 10.1016/j.jacasi.2022.04.007

**Published:** 2022-09-06

**Authors:** Masanori Yamamoto, Toshiaki Otsuka, Tetsuro Shimura, Ryo Yamaguchi, Yuya Adachi, Ai Kagase, Takahiro Tokuda, Satoshi Tsujimoto, Yutaka Koyama, Fumiaki Yashima, Norio Tada, Toru Naganuma, Masahiro Yamawaki, Futoshi Yamanaka, Shinichi Shirai, Kazuki Mizutani, Minoru Tabata, Hiroshi Ueno, Kensuke Takagi, Yusuke Watanabe, Kentaro Hayashida, OCEAN-TAVI Investigators

**Affiliations:** a Department of Cardiology, Toyohashi Heart Center, Toyohashi, Japan; b Department of Cardiology, Nagoya Heart Center, Nagoya, Japan; c Department of Hygiene and Public Health, Nippon Medical School, Tokyo, Japan; d Center for Clinical Research, Nippon Medical School Hospital, Tokyo, Japan; e Department of Cardiology, Saiseikai Utsunomiya Hospital, Tochigi, Japan; f Department of Cardiology, Keio University School of Medicine, Tokyo, Japan; g Department of Cardiology, Sendai Kosei Hospital, Sendai, Japan; h Department of Cardiology, New Tokyo Hospital, Chiba, Japan; i Department of Cardiology, Saiseikai Yokohama City Eastern Hospital, Yokohama, Japan; j Department of Cardiology, Shonan Kamakura General Hospital, Kanagawa, Japan; k Department of Cardiology, Kokura Memorial Hospital, Kokura, Japan; l Department of Cardiology, Kindai University, Osaka, Japan; m Department of Cardiovascular Surgery, Tokyo Bay Urayasu-Ichikawa Medical Center, Chiba, Japan; n Department of Cardiology, Toyama University Hospital, Toyama, Japan; o Department of Cardiology, Ogaki Municipal Hospital, Gifu, Japan; p Department of Cardiology, Teikyo University School of Medicine, Tokyo, Japan

**Keywords:** clinical outcome, late bleeding, transcatheter aortic valve replacement, AF, atrial fibrillation, BARC, Bleeding Academic Research Consortium, CFS, clinical frailty scale, DAPT, dual antiplatelet therapy, GI, gastrointestinal, NYHA, New York Heart Association, OAC, oral anticoagulant, OR, odds ratio, PCI, percutaneous coronary intervention, SPAT, single antiplatelet therapy, TAVR, transcatheter aortic valve replacement

## Abstract

**Background:**

Data regarding the incidence, predictive factors, and clinical outcomes of post-transcatheter aortic valve replacement (TAVR) bleeding is limited in the Asian cohort.

**Objectives:**

This study sought to assess the predictors and prognostic impact of post-TAVR late bleeding.

**Methods:**

This study used the Japanese multicenter registry data to analyze 2,518 patients (mean age: 84.3 ± 5.2 years) who underwent TAVR. Late bleeding was defined as any postdischarge bleeding events after TAVR. Baseline characteristics, predictive factors, and clinical outcomes including death and rehospitalization were assessed in patients with and without late bleeding events.

**Results:**

The cumulative incidence rate of all and major late bleeding and ischemic stroke were 7.4%, 5.2%, and 3.4%, respectively, 3 years after TAVR. The independent predictive factors of late bleeding were low platelet count, high score (≥4) on the clinical frailty scale, and a New York Heart Association functional class III/IV. The cumulative mortality rates up to 3 years were significantly higher in patients with late bleeding than in those without bleeding (*P <* 0.001). The multivariate Cox regression analysis revealed that late bleeding, included as a time-varying covariate in the model, was associated with an increased risk of mortality following TAVR (HR: 5.63; 95% CI: 4.28-7.41; *P <* 0.001).

**Conclusions:**

Late bleeding after TAVR was not a rare complication, and it significantly increased long-term mortality. It should be carefully managed, especially when it is predictable in the high-risk cohort, and efforts should be taken to reduce bleeding complications even after a successful procedure.

With the development of clinical devices and evidences, transcatheter aortic valve replacement (TAVR) has been broadly indicated for patients with degenerative severe aortic stenosis.^1^, ^2^, ^3^ Considering the widespread use of the TAVR procedure, late adverse clinical events need to be elucidated separately from the procedural complications. Postdischarge bleeding after percutaneous coronary intervention (PCI) is a significant clinical issue because it is found to be related to the increased risk of readmission and mortality during the follow-up period.^4^, ^5^, ^6^ The TAVR cohort is generally composed of patients with specific features such as old age, high frailty, and multiple comorbidities.^7^^,^^8^ The antithrombotic regimen after the procedure should be carefully decided according to the risk-benefit balance to avoid excessive bleeding and ischemic events, especially in the high-risk subsets of bleeding events. However, there are only a few studies describing late bleeding complications after TAVR.^9^^,^^10^ Furthermore, no data exist for this investigation in the Asian cohort. Therefore, this study aimed to clarify the incidence, timing, cause, predictive factors, and clinical outcomes related to late bleeding events in patients who had undergone TAVR, using a Japanese multicenter data.

## Methods

### Study population

Data were extracted from the ongoing prospective Japanese multicenter OCEAN-TAVI (Optimized Transcatheter Valvular Intervention-Transcatheter Aortic Valve Implantation) registry.^7^^,^^8^ A total of 2,588 patients scheduled for TAVR were enrolled in this registry from October 1, 2013, to May 31, 2017. Of these, 70 patients who died during the index hospital admission were excluded from the initial inclusion. The data of the remaining 2,518 patients were retrospectively examined in terms of postdischarge bleeding events after TAVR. Conventional data collection included baseline patient characteristics, laboratory data, echocardiographic data, procedural variables, and clinical outcomes in terms of mortality, rehospitalization, postdischarge bleeding, stroke, and so on. Data reported on the internet-based system were checked via self-auditing through websites. Data committee members also confirmed the completeness and consistency of the database and regularly sent queries to each center if necessary. This study protocol was approved by the local institutional review board and was registered with the University Hospital Medical Information Network (UMIN000020423). Written informed consent was obtained from all patients before they underwent TAVR.

### Data definition

The procedure of TAVR has been previously reported.^7^^,^^8^ After TAVR, dual antiplatelet therapy (DAPT) is usually recommended for 3-6 months, followed by single antiplatelet therapy (SAPT) indefinitely. Most of the patients who underwent TAVR and/or PCI were prescribed DAPT comprising aspirin combined with thienopyridine. The distribution of antithrombotic therapy prescribed at discharge was categorized into 4 groups: 1) none or SAPT group (n = 545); 2) DAPT group (n = 1,361); 3) oral anticoagulants (OACs) and none or SAPT group (n = 577); and 4) OAC and DAPT group (n = 35). Patients who were administered OACs were not included in groups 1 and 2, but were included in groups 3 and 4. The antithrombotic therapy at the time of late bleeding events was also analyzed. The dynamic changes of the antithrombotic drug regimen from discharge to the date of the late bleeding event were classified as either maintained (no change of antithrombotic therapy), de-escalated (discontinuation of SAPT/DAPT or OAC), or escalated (addition of SAPT/DAPT or OAC).

Procedural complications and postdischarge stroke events were evaluated according to the Valve Academic Research Consortium-2 criteria.^11^ Late bleeding was defined as any postdischarge bleeding events, and the exact period from the procedure to the date of late bleeding was calculated. The Bleeding Academic Research Consortium (BARC) criteria were additionally adopted to define the severity of late bleeding events.^12^ Major and minor late bleeding events were defined as higher than BARC type 3a and lower than BARC type 2, respectively. Data regarding transfusion use, hemoglobin drop, hemodynamic status, and presumed mortality caused by bleeding during hospitalization were checked to match the BARC-2 bleeding criteria. These reports were extracted from the medical records of each treating hospital and clinic. The cause of bleeding was classified as gastrointestinal (GI) bleeding, hemorrhagic stroke, trauma, cancer, and others, which included unspecified or clinically unknown causes.

### Statistical analysis

All statistical analyses were performed using SPSS software (version 22, IBM Corp), R software packages (version 3.0.1, R Development Core Team), and Stata 14 (Stata Corp). Continuous variables are expressed as mean ± SD and median (IQR). Differences were tested using the unpaired Student’s *t-*test or Mann-Whitney *U* test depending on the variable distribution. Baseline and procedural outcomes were compared between late bleeding and non–late bleeding groups. Univariate and multivariate logistic regression analyses were performed to obtain the odds ratio (OR) of each variable for predicting late bleeding events. A multivariate analysis was performed using the baseline clinical characteristics with a univariate *P* value of <0.10 and other important variables such as age, sex, and procedural bleeding complications to examine their independent associations to late bleeding. The classification and regression tree analysis is an empirical and statistical method to create decision rules based on data rather than speculation and to create the risk stratification model.^13^ The classification and regression tree analysis identified the optimal threshold of platelet count. The clinical frailty scale (CFS) reflects the semiquantitative assessment of a patient’s frailty, and its cutoff in the present study was ≥4. Values above this threshold indicated frailty.^8^ The univariate and multivariate Cox regression analyses were performed to determine the HR for predicting long-term mortality. A multivariate analysis was performed using the baseline clinical characteristics with a univariate *P* value of <0.10 and other important variables such as age, hypertension, and renal function evaluated by estimated glomerular filtration ratio to examine their independent associations to clinical outcomes. The late bleeding event was included as a time-varying covariate in the model. In the multivariate Cox model, we first entered the potential confounders for mortality as explanatory variables. The Kaplan–Meier curve was used to estimate the cumulative mortality, bleeding, and ischemic stroke events. The differences of each category and its subgroups were assessed with the log-rank test. In addition, pairwise analyses were performed to compare mortality rates among the no bleeding, minor bleeding, and major bleeding groups using the log-rank test with Bonferroni correction. All statistical tests were 2-sided, and a *P* value of <0.05 was considered statistically significant.

## Results

### Baseline and procedural characteristics of patients with late bleeding

The patients’ baseline characteristics are shown in Table 1. The proportion of male patients was 30.5%, and the mean age of all patients was 84.3 ± 5.2 years. The frequency of CFS ≥4, New York Heart Association (NYHA) functional class III/IV, liver disease, and active cancer was significantly higher in the late bleeding group than in the no late bleeding group (all *P <* 0.05). The classification and regression tree analysis identified that the optimal platelet count threshold was 14.9 × 10^4^/μL for predicting the risk of late bleeding events after TAVR. The existence of atrial fibrillation (AF) was observed in 508 patients, while 15.9% of AF patients were not administered OACs (n = 81 of 508). The patients with AF, who were prescribed OACs, exhibited a trend toward a higher incidence of late bleeding compared with those who were not prescribed OACs (22.1% vs 16.7%; *P =* 0.098). The prevalence of gastric acid–suppressive agents usage showed no significant differences between the 2 groups *(P =* 0.42). In addition, the distributions of gastric acid–suppressive agents did not differ in patients with GI bleeding and no GI bleeding *(P =* 0.38; data not shown). Procedural variables and complications are presented in Table 2. There were no significant differences in the hospital and intensive care unit stay, type of valve, approach route, and periprocedural complications, including any bleeding complications between the 2 groups.

**Table 1:** Baseline Patient Characteristics

	Overall (N = 2,518)	Late Bleeding (n = 140)	No Late Bleeding (n = 2,378)	*P* Value
Baseline clinical characteristics				
Age, y	84.3 ± 5.2	84.6 ± 5.3	84.3 ± 5.2	0.59
Male	769 (30.5)	49 (35.0)	720 (30.3)	0.26
Weight, kg	50.2 ± 10.2	50.7 ± 10.0	50.2 ± 10.3	0.56
BSA, m^2^	1.43 ± 0.17	1.45 ± 0.18	1.43 ± 0.17	0.20
BMI, kg/m^2^	22.2 ± 3.6	22.1 ± 3.3	22.2 ± 3.6	0.72
CFS ≥4	1,492 (59.3)	97 (69.3)	1,395 (58.7)	0.013
NYHA functional class III/IV	1,272 (50.5)	88 (62.9)	1,184 (49.8)	<0.001
Hypertension	1,934 (76.8)	106 (75.7)	1,828 (76.9)	0.76
Diabetes	542 (21.5)	30 (21.4)	512 (21.5)	>0.99
Pre-existing AF	502 (19.9)	36 (25.7)	466 (19.6)	0.078
Pre-existing AF with OACs	427 (17.0)	31 (22.1)	396 (16.7)	0.093
Coronary artery disease	915 (36.3)	55 (39.3)	860 (36.2)	0.46
Chronic kidney disease	1,749 (69.5)	101 (72.1)	1,648 (69.3)	0.51
Peripheral artery disease	353 (14.0)	25 (17.9)	328 (13.8)	0.21
Pulmonary disease	590 (23.4)	42 (30.0)	548 (23.0)	0.065
Liver disease	75 (3.0)	10 (7.1)	65 (2.7)	<0.01
Active cancer	124 (4.9)	13 (9.3)	111 (4.7)	0.024
Previous stroke	285 (11.3)	18 (12.9)	267 (11.2)	0.58
Prior CABG	159 (6.3)	7 (5.0)	152 (6.4)	0.72
STS score, %	6.5 (4.5-9.4)	8.1 ± 6.2	8.1 ± 6.6	>0.99
Laboratory data				
Serum Na, mEq/L	139.8 ± 4.5	139.4 ± 3.5	139.8 ± 4.6	0.26
Serum K, mEq/L	4.3 ± 0.5	4.3 ± 0.5	4.3 ± 0.5	0.65
Serum albumin, g/dL	3.8 ± 0.5	3.7 ± 0.4	3.8 ± 0.5	0.11
Creatinine, mg/dL	1.0 ± 0.5	1.1 ± 0.6	1.0 ± 0.5	0.40
eGFR, mL/min/1.73 m^2^	51.6 ± 19.4	51.4 ± 20.7	51.6 ± 19.3	0.93
Hemoglobin, mg/dL	11.3 ± 1.7	11.1 ± 1.7	11.3 ± 1.7	0.27
Platelet count, ×10^4^ /μL	18.3 ± 6.8	16.5 ± 6.1	18.4 ± 6.8	<0.001
Gastric acid–suppressive agents				
Proton pomp inhibitor	1,614 (64.1)	97 (79.3)	1,517 (63.8)	
Histamine-2 blocker	148 (5.9)	7 (5.0)	141 (5.9)	0.42
No drug agents	756 (30.0)	36 (25.7)	720 (30.3)	
Antithrombotic therapy				
None or SAPT	545 (21.6)	22 (15.7)	523 (22.0)	
DAPT	1,361 (54.1)	76 (54.3)	1,285 (54.0)	0.040
OAC + none or SAPT	577 (22.9)	37 (26.4)	540 (22.7)	
OAC + DAPT	35 (1.4)	5 (14.3)	30 (1.3)	
Echocardiographic data				
AVA, cm^2^	0.63 ± 0.17	0.63 ± 0.18	0.63 ±0.17	0.94
Indexed AVA, cm^2^/m^2^	0.44 ± 0.12	0.44 ± 0.11	0.47 ± 0.12	0.57
Peak velocity, m/s	4.6 ± 0.79	4.6 ± 0.73	4.6 ± 0.79	0.39
Peak gradient, mm Hg	86.1 ± 29.4	87.3 ± 26.8	86.0 ± 29.6	0.62
Mean gradient, mm Hg	50.7 ± 18.3	51.2 ± 17.2	50.6 ± 18.3	0.70
LVEF, %	59.3 ± 12.6	59.0 ± 12.9	59.3 ± 12.6	0.80

Values are mean ± SD, n (%), or median (IQR).

AF = atrial fibrillation; AVA = aortic valve area; BSA = body surface area; BMI = body mass index; CABG = coronary artery bypass graft; CFS = clinical frailty scale; DAPT = dual antiplatelet therapy; eGFR = estimated glomerular filtration rate; LVEF = left ventricle ejection fraction; NYHA = New York Heart Association; OAC = oral anticoagulant; SAPT = single antiplatelet therapy; STS = Society of Thoracic Surgeons.

**Table 2:** Procedural Patient Characteristics

	Overall (N = 2,518)	Late Bleeding (n = 140)	No Late Bleeding (n = 2,378)	*P* Value
Procedural variables				
Procedure time, min	78.2 ± 39.1	83.7 ± 39.9	77.9 ± 39.0	0.09
Fluoroscopy time, min	20.7 ± 11.2	20.7 ± 9.9	20.7 ± 11.3	0.96
Contrast media, mL	114.2 ± 58.4	115.0 ± 50.3	114.2 ± 58.9	0.86
Length of intensive care unit, d	1.0 (1.0-2.0)	1.0 (1.0-2.0)	1.0 (1.0-2.0)	0.41
Length of hospital stay, d	10.0 (7.0-15.0)	9.5 (10.0-20.0)	10 (8.0-16.0)	0.85
Type of valve				
Balloon expandable valve	2,187 (86.9)	127 (90.7)	2,060 (86.6)	0.20
Self-expandable valve	331 (13.1)	13 (9.3)	318 (13.4)	
Approach route				
Transfemoral	2,155 (85.6)	122 (87.1)	2,033 (85.5)	0.71
Nontransfemoral	363 (14.4)	18 (12.9)	345 (14.5)	
Procedural complications				
30-d mortality	6 (0.2)	0 (0.0)	6 (0.3)	>0.99
New onset AF	93 (3.7)	4 (2.9)	89 (3.7)	0.60
Acute coronary occlusion	23 (0.9)	2 (1.4)	21 (0.9)	0.37
Disabling stroke	32 (1.3)	3 (2.1)	29 (1.2)	0.42
Hemorrhagic stroke	4 (0.2)	0 (0)	4 (0.2)	0.80
Acute kidney injury	243 (9.7)	10 (7.1)	233 (9.8)	0.38
Major vascular complications	97 (3.9)	7 (5.0)	90 (3.8)	0.49
Life-threatening and major bleeding	349 (13.9)	18 (12.9)	331 (13.9)	0.42
Minor bleeding	224 (8.9)	15 (10.7)	209 (8.8)	0.26
None-trivial PVL	1,619 (64.3)	83 (59.3)	1,536 (64.6)	
Mild PVL	854 (33.1)	52 (37.1)	802 (33.7)	0.16
Moderate-severe PVL	45 (1.8)	5 (3.6)	40 (1.7)	
Need for any cardiac surgery	13 (0.5)	2 (1.4)	11 (0.5)	0.16

Values are mean ± SD, median (IQR), or n (%).

AF = atrial fibrillation; PVL = paravalvular aortic regurgitation.

### Incidence, timing, and causes of late bleeding events after TAVR

**Figure 1:**
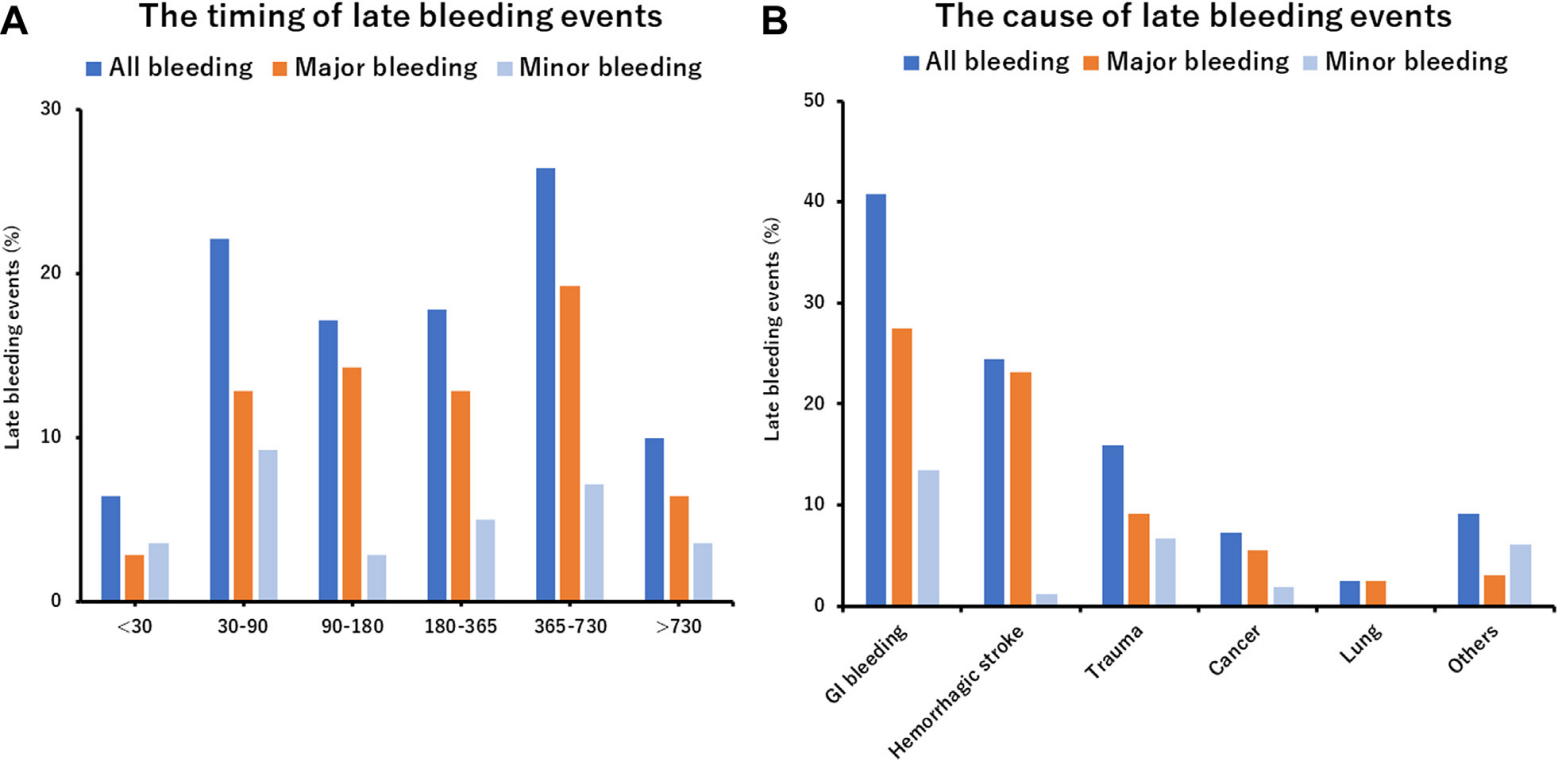
Timing and Causes of Late Bleeding Events After TAVR **(A)** The phase of late bleeding evens after transcatheter aortic valve replacement (TAVR) was calculated. The late bleeding events occurred from early to late phase after TAVR. **(B)** The detailed cause of bleeding information was investigated. The main cause of late bleeding was of gastrointestinal (GI) origin. The second cause of bleeding was hemorrhagic stroke that mainly associated with major bleeding. The other causes of bleeding were trauma, cancer, lung, and other organs including the eye, nose, and the genitourinary system.

**Figure 2:**
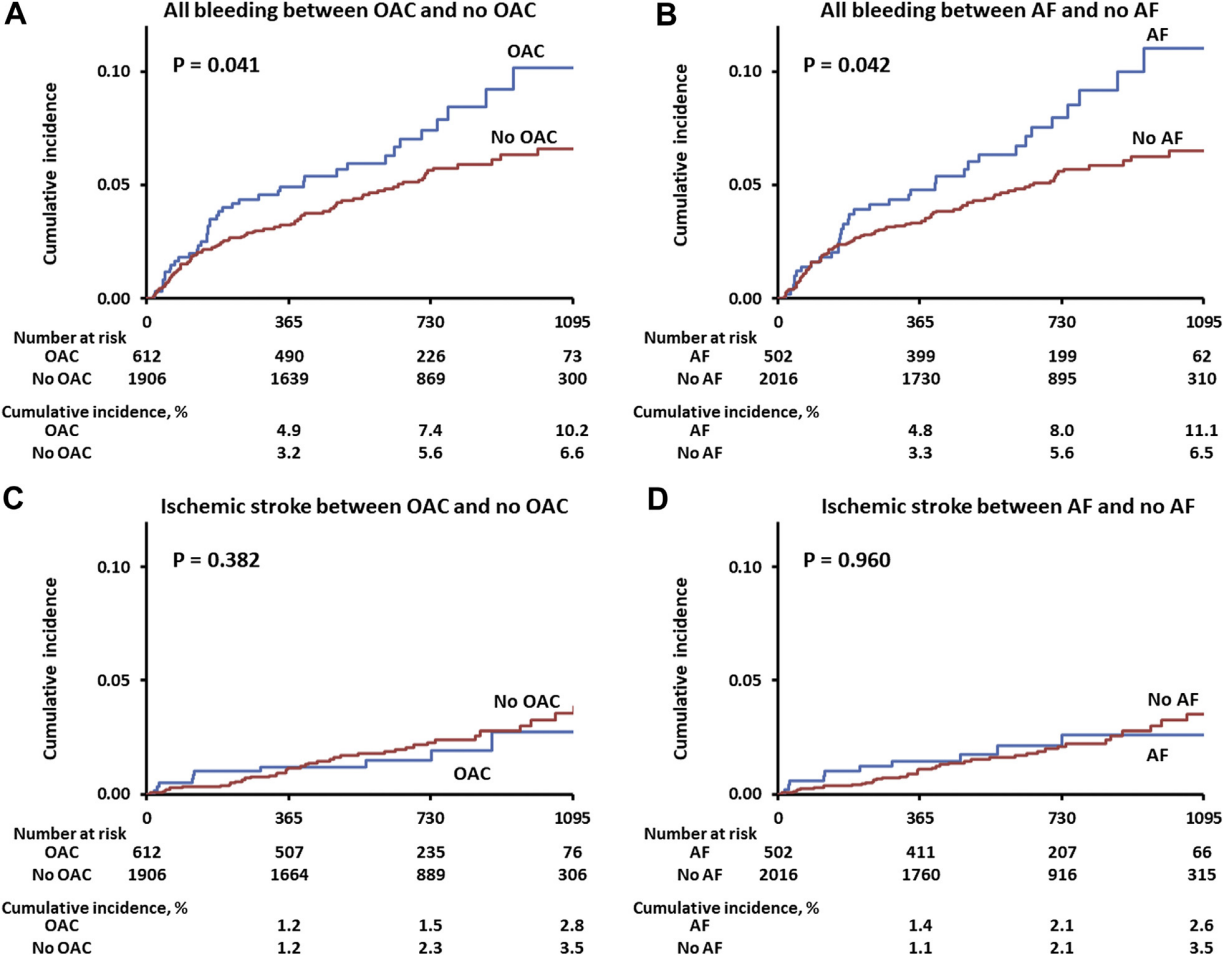
Subgroup Events Analysis of OAC and AF After TAVR **(A)** The subgroup analysis indicated the increased risk of late bleeding events in patients with oral anticoagulant (OAC) use compared with in those without OAC use. Higher bleeding risk was found in the elderly cohort with transcatheter aortic valve replacement (TAVR) that was being administered OACs. **(B)** The increased late bleeding events were found in patients with atrial fibrillation (AF) rather than in those without AF. **(C)** In contrast to the late bleeding events, the subgroup analysis indicated the similar rate of ischemic stroke between the OAC and no OAC groups. **(D)** The rate of ischemic stroke was similar between the AF and no AF groups.

**Figure 3:**
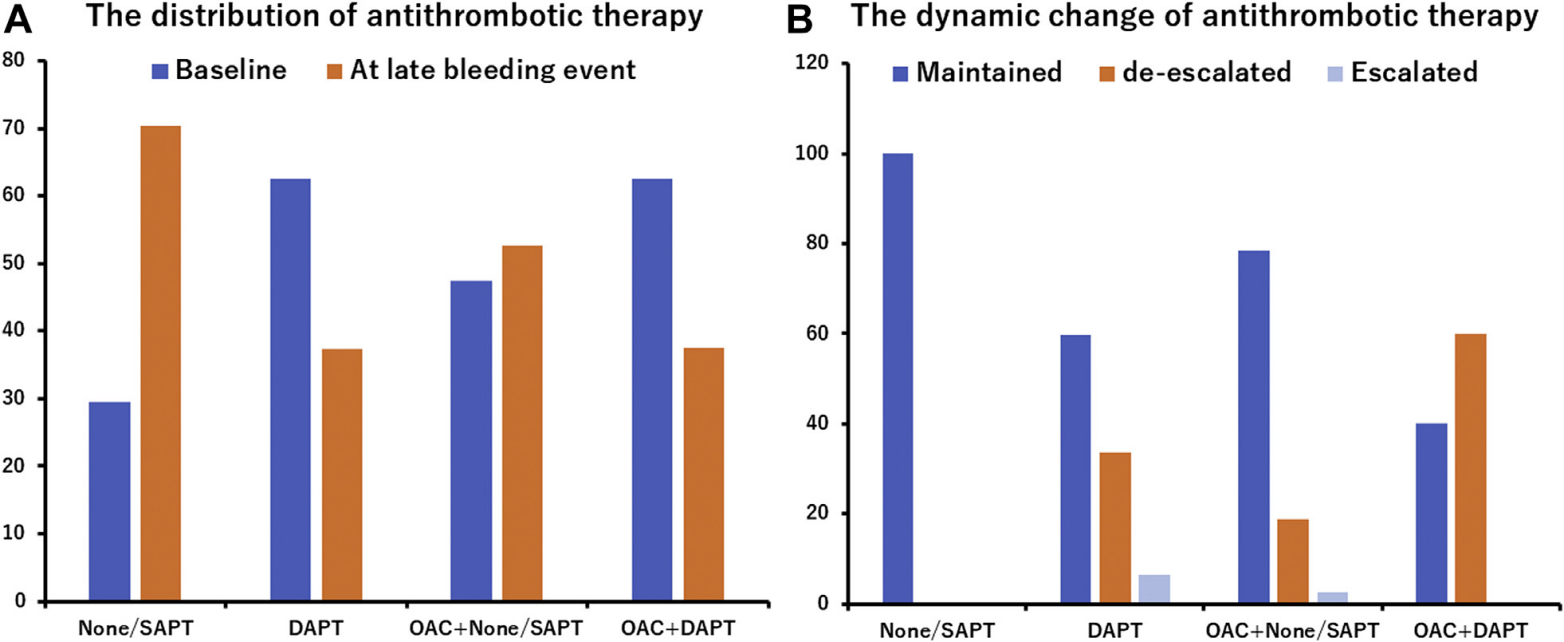
Distribution and Dynamic Change of Antithrombotic Therapy at Bleeding **(A)** Antiplatelet therapy tended to de-escalate, whereas oral anticoagulant (OAC) therapy was maintained at the time of late bleeding. **(B)** The physicians tried to avoid an aggressive antithrombotic drug regimen to reduce the risk of late bleeding, thus many patients de-escalated the dose of antithrombotic therapy at the timing of late bleeding. DAPT = dual antiplatelet therapy; SAPT = single antiplatelet therapy.

The median follow-up duration was 693 (IQR: 389.0-871.3) days, and the 1-year clinical follow-up rate was 99.5%. Overall, late bleeding and rehospitalization in conjunction with bleeding occurred in 140 and 122 patients, respectively. The timing of late bleeding events after TAVR is presented in Figure 1A. Among the patients who experienced late bleeding, the event occurred in 6.4% of patients within 30 days, 22.1% at 30-60 days, 17.9% at 90-180 days, 17.1% at 180-365 days, 26.4% at 365-730 days, and 10% at >730 days, following TAVR. The causes of bleeding are also presented in Figure 1B, which include GI bleeding (40.7%); hemorrhagic stroke (25.7%); trauma (15.0%); cancer (7.2%); lung bleeding (2.4%); and bleeding from other organs including the eye, nose, and genitourinary system, as well as unknown causes (9.0%). The cumulative incidence of all, major, and minor late bleeding and ischemic stroke were 7.4%, 5.2%, 2.5%, and 3.4%, respectively, at 3 years (Central Illustration). The all and major bleeding risks were significantly higher than the risk of stroke events (log-rank test; *P <* 0.001). The incidences of late bleeding events were compared between the subgroup of patients with or without OAC and AF (Figures 2A and 2B). The rates of major bleeding were significantly higher in patients with OACs than in those without OACs, and in patients with AF than in those without AF *(P =* 0.041 and *P =* 0.042, respectively). The rates of ischemic stroke did not differ significantly between the OAC and no OAC groups (Figure 2C) and between the AF and no AF groups (Figure 2D). The incidence of bleeding complications and ischemic stroke events were also compared in patients with or without OACs and AF (Supplemental Figure 1). The major bleeding complications were significantly higher than those of ischemic stroke events in patients regardless of OAC use and AF (all *P <* 0.05, raw *P* value by log-rank test). The distribution of antithrombotic therapy at the late bleeding event was significantly different from that of baseline therapy (Figure 3A). The dynamic change of antithrombotic therapy from discharge to the date of the late bleeding event is shown in Figure 3B. The detailed information concerning the dynamic change of antithrombotic therapy is shown in Table 3. At the time of late bleeding, 33.8% (n = 26 of 77) of patients with DAPT at discharge were de-escalated to none/SAPT, whereas 93.0% of patients (n = 3 of 42) with OAC maintained the medication from discharge to the time of late bleeding. In the OAC group, the prevalence of AF was 76.2% at discharge and 68.2% at the timing of late bleeding.

**Central Illustration:**
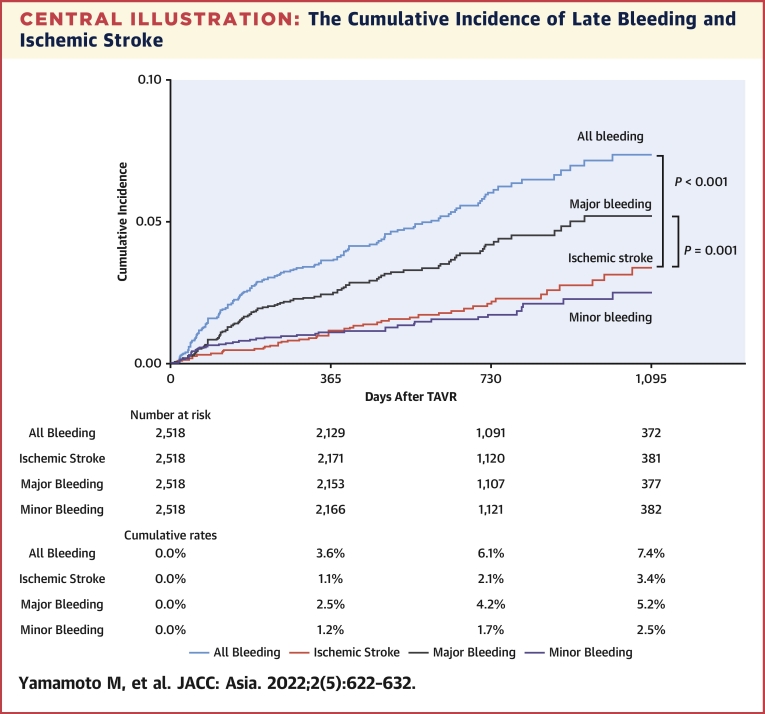
The Cumulative Incidence of Late Bleeding and Ischemic Stroke The incidences of all late bleeding events were higher than ischemic stroke events after transcatheter aortic valve replacement (TAVR) (log-rank test; *P <* 0.001). The major bleeding events were also significantly higher than ischemic stroke events (log-rank test; *P =* 0.001). Physicians should pay attention for relatively higher bleeding complications in the elderly cohort with TAVR.

**Table 3:** Detailed Information Concerning the Dynamic Change of Antithrombotic Therapy

	None/SAPT	DAPT	OAC + None/SAPT	OAC + DAPT
Late bleeding patients (n = 140)	21	77	37	5
De-escalation of SAPT/DAPT	(-)	26	5	2
De-escalation of OAC	(-)	(-)	2	1
Escalation of SAPT	(-)	(-)	1	(-)
Escalation of OAC	(-)	5	(-)	(-)

	No OAC Group	OAC Group
Late bleeding patients (n = 140)	98	42
De-escalation of SAPT/DAPT	26	7
De-escalation of OAC	(-)	3
Escalation of SAPT	(-)	1
Escalation of OAC	5	(-)
AF status at discharge	(-)	32/42 (76.2)
AF status at late bleeding event	(-)	30/44 (68.2)

Values are n. (-) indicates no change.

Abbreviations as in Table 1.

### The predictive factors of late bleeding

The results of the univariate and multivariate analyses for the association between the risk of late bleeding and clinical findings are shown in Supplemental Table 1 and Table 4. Pre-existing AF alone was not related to the increased risk of late bleeding in the univariate model (OR: 1.33; 95% CI: 0.90-1.96; *P =* 0.16). The independent predictive factors of late bleeding were high CFS (≥4) (OR: 1.55; 95% CI: 1.05-2.27; *P =* 0.015), NYHA functional class III/IV (OR: 1.58; 95% CI: 1.09-2.27; *P =* 0.015), and low platelet count (OR: 1.94; 95% CI: 1.36-2.77; *P <* 0.001).

**Table 4:** Multivariate Analysis for the Association Between Late Bleeding and Clinical Findings

Explanatory Variables	Multivariate Analysis		
	OR	95% CI	*P* Value
Baseline characteristics			
Age (per 1 y)	1.01	0.98-1.04	0.60
Male	1.25	0.86-1.82	0.25
High CFS( ≥4)	1.55	1.05-2.28	0.027
NYHA functional class III/IV (for I/II)	1.58	1.09-2.27	0.015
Pulmonary disease	1.41	0.96-2.07	0.084
Liver disease	1.93	0.92-4.07	0.084
Active cancer	1.87	0.98-3.54	0.057
Low platelet count (<14.9 × 10^4^/μL)	1.94	1.36-2.77	<0.001
Procedural bleeding complications	0.92	0.61-1.40	0.70

Abbreviations as in Table 1.

### Clinical outcomes in patients with late bleeding

**Figure 4:**
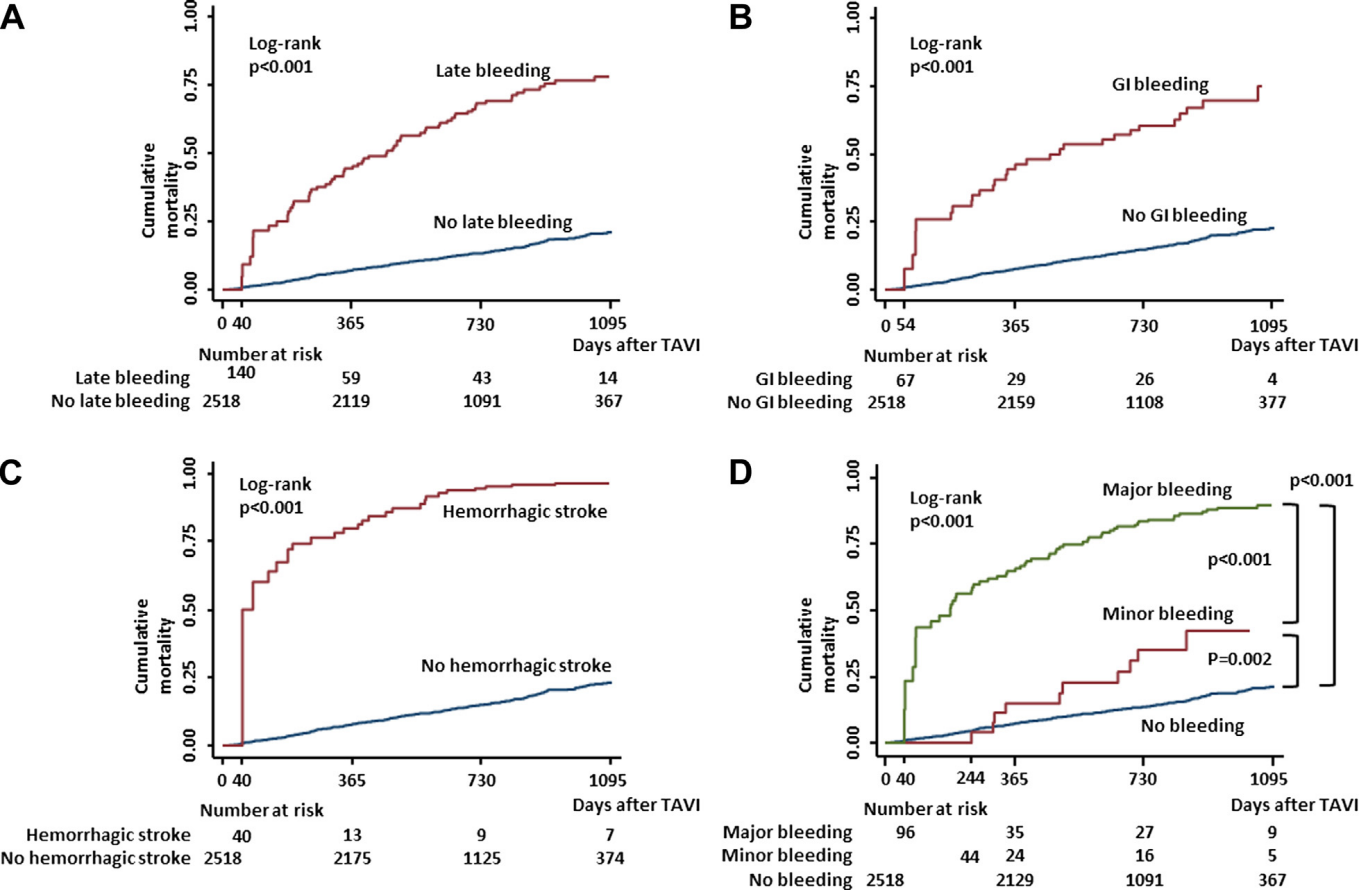
Subgroup Kaplan-Meier Curves Showing Bleeding and No Bleeding **(A)** Follow-up was initiated from the day when TAVR was performed in the no bleeding event group and from the day when bleeding event occurred in the bleeding group. The worse prognosis of late bleeding event was observed. **(B)** The subgroup analysis showed significant increase in mortality in patients with gastrointestinal bleeding than in those without gastrointestinal bleeding. **(C)** The subgroup analysis showed significant increase in mortality in patients with hemorrhagic stroke than in those without hemorrhagic stroke. **(D)** A significant increase in mortality was found among patients with major and minor bleeding than among those without bleeding events. Abbreviations as in Figure 1.

The Kaplan-Meier curves show the significant differences in terms of mortality between the late bleeding and no late bleeding groups after TAVR (Figure 4A). The cumulative mortality rates in the late bleeding group were significantly higher than those of the no late bleeding group (log-rank test; *P <* 0.001). The subgroup analysis for the cumulative mortality of GI bleeding, hemorrhagic stroke, and BARC-defined minor and major bleeding are also shown in Figures 4B to 4D. Significant increases in mortality rate were found in patients with GI bleeding, hemorrhagic stroke, and major bleeding compared with in those without these bleeding events (log-rank test, all *P <* 0.001). In addition, a significant increase in mortality was found among those with minor bleeding than those without bleeding events *(P =* 0.002). The results of univariate and multivariate analysis for predicting late mortality are presented in the Supplemental Table 2 and Table 5. The multivariate Cox regression analysis revealed that late bleeding was independently associated with increased risk for late mortality after TAVR (HR: 5.40; 95% CI: 4.09-7.13; *P <* 0.001). The other subgroup analysis also showed a significantly increased risk of mortality in patients with GI bleeding (HR: 3.38; 95% CI: 2.26-5.05; *P <* 0.001), hemorrhagic stroke (HR: 10.5; 95% CI: 6.85-16.0; *P <* 0.001), minor bleeding (HR: 2.78; 95% CI: 1.52-5.08; *P =* 0.001), and major bleeding (HR: 6.81; 95% CI: 5.02-9.26; *P <* 0.001).

**Table 5:** Cox Regression Analysis for the Association Between Late Bleeding and Clinical Outcomes

	Multivariate Analysis^a^		
	HR^b^	95% CI	*P* Value
Types of bleeding			
All late bleeding (for no all late bleeding)	5.40	4.09-7.13	<0.001
GI bleeding (for no GI bleeding)	3.38	2.26-5.05	<0.001
Hemorrhagic stroke (for no hemorrhagic stroke)	10.5	6.85-16.0	<0.001
Bleeding severity (reference as no bleeding)			
Minor bleeding	2.78	1.52-5.08	0.001
Major bleeding	6.81	5.02-9.26	<0.001

Abbreviations as in Table 1.

a Adjusted for sex, age, body mass index, diabetes, hypertension, eGFR, AF, pre-existing CABG surgery, peripheral artery disease, stroke, pulmonary disease, coronary artery disease, liver disease, STS, CFS ≥4, NYHA functional class ≥III, active cancer, and low platelet count.

b Calculated using Cox proportional hazards models with bleeding event as time-varying covariates.

## Discussion

In the current study, the incidences of late bleeding and major bleeding after TAVR were 7.4% and 5.2% in the Asian cohort, respectively. The significant predictive factors of late bleeding were low platelet count, CFS ≥4 (patients with frailty), and NYHA functional class III/IV. The occurrence of late bleeding after TAVR was independently associated with an increased risk of mortality following TAVR. In addition, the rates of major late bleeding were significantly higher than those of ischemic strokes. These results were not attenuated for patients with OAC use or AF. Therefore, physicians should pay attention to reducing the bleeding complication even after a successful TAVR procedure.

The all and major bleeding risks were significantly higher than the risk of stroke events. Interestingly, the subgroup analysis of patients in the OAC and no OAC groups (AF and no AF groups) showed different bleeding and ischemic stroke rates after TAVR. The rates of major bleeding were significantly higher in patients with OACs than in those without OACs, and in patients with AF than in those without AF. Although the rates of ischemic stroke did not differ significantly between the OAC and no OAC groups and AF and no AF groups. The major bleeding complications were significantly higher than those of ischemic stroke events in patients regardless of OAC use and AF, which reflects the higher bleeding complications because of the OAC prescription. Whereas OACs are effective in preventing cardiogenic stroke, the clinical dilemma between the risk-benefit balance of bleeding and ischemic events was highlighted in patients with OACs and AF. Caution is warranted for relatively higher bleeding complications in the elderly TAVR cohort that was being administered OACs.

Previous investigations revealed that the incidences of late bleeding after TAVR were 5.9% and 11.3%, respectively.^9^, ^10^ The main causes of bleeding were GI bleeding, hemorrhagic stroke, trauma, and active cancer. These trends were confirmed by the previous TAVR data from Western countries; thus, there are no ethnic differences concerning the risk of late bleeding after TAVR.^9^, ^10^ While the percentage of late bleeding and rehospitalization after TAVR were similar or relatively higher than that after PCI, the time distribution of late bleeding was thought to be different.^4^, ^5^, ^6^^,^^14^, ^15^ The incidence of late bleeding in the early phase (within 30 days) after TAVR and PCI was 6.4% and approximately 10%-20%, respectively.^4^, ^5^, ^6^^,^^14^, ^15^ While almost all patients received DAPT after PCI to prevent stent thrombosis, the antithrombotic regimen was varied and less aggressive in the TAVR cohort. Furthermore, 20% of the patients either required no antithrombotic therapy or received SAPT alone. The vascular and bleeding complications during TAVR were managed in the index hospital admission, which resulted in longer hospital stays.^16^ Therefore, the rates of postdischarge bleeding within 30 days were lower and late bleeding events occurred after >30 days in the TAVR cohort.

The significant predictive factors of late bleeding included low platelet count, CFS ≥4 (patients with frailty), and NYHA functional class III/IV. These variables predicting late bleeding in our study were considerable factors used to estimate the cumulative incidence of future bleeding events.^17^, ^18^, ^19^ and postdischarge bleeding after PCI as well.^20^, ^21^ Considering the risk of late bleeding in patients on antiplatelet therapy, it is important to evaluate the dynamic change of the antithrombotic regime and drug information at the time of late bleeding. Although the data regarding serial change of drug information was hard to collect, the antithrombotic therapy was retrospectively analyzed in detail in patients with late bleeding. One-third of patients with DAPT at discharge were reduced to none/SAPT status at the time of the late bleeding event, whereas more than 90% of patients continued the OAC medication from the time of discharge until the late bleeding event. These differences may explain the different results in the multivariate analysis predicting late bleeding after TAVR. Contrarily, patient age was not a risk factor for late bleeding in our TAVR cohort.^17^, ^18^, ^19^, ^20^, ^21^ The patients’ average age was approximately 85 years, and the majority of patients were octogenarians in this study. In such a specific cohort, age differences might be attenuated to predict the risk of bleeding. Additionally, the frailty status evaluated by CFS was a significant predictive factor of late bleeding. Elderly patients with frailty have fragile tissues and may fall while walking, thereby causing trauma.^22^^,^^23^ We had previously revealed that a higher CFS grade was significantly related to poor clinical outcomes after TAVR.^8^ The CFS assessment allows us to stratify not only the mortality risk but also the high late bleeding risk after TAVR.

The time-adjusted multivariable Cox regression analysis suggested that the occurrence of late bleeding events was independently associated with poor prognosis after TAVR. The cumulative mortality was significantly higher in patients with late bleeding than in those without late bleeding events. This result did not change in the subgroup analysis according to the bleeding severity as defined by the BARC criteria, GI bleeding, and hemorrhagic stroke following TAVR. Antithrombotic therapy is among the key factors for estimating bleeding events.^17^^,^^18^ Short-term DAPT prescription after coronary stenting should be taken into account when considering the risk-benefit balance of bleeding and thrombotic events.^24^ Patients on OAC medication should be advised to discontinue early the combination of SAPT and DAPT therapy because of their increased bleeding risk after PCI or surgery.^25^^,^^26^ Although about 75% of the patients in this study were prescribed DAPT in the no OAC group, a recent randomized study has demonstrated low rates of bleeding and similar rates of stroke events in patients prescribed SAPT instead of DAPT after TAVR.^27^ A recent randomized trial also demonstrated high bleeding rate and increased mortality in patients who were prescribed OACs, compared with in those receiving antiplatelet therapy after TAVR.^28^ This cohort comprised patients without an established indication for OACs, indicating that the routine OAC prescription after TAVR is unnecessary in daily practice. Although the optimal antithrombotic therapy after TAVR is still controversial, an aggressive antithrombotic drug regimen may be harmful and early reduction or discontinuation of antithrombotic therapy may be beneficial for a subset of patients with a high risk for bleeding.

### Study limitations

This study involved a large number of patients but randomization was not applied. All late bleeding events were collected through self-reports from individual centers. The number of minor late bleeding events was relatively less than those of major bleeding events. Although bleeding with readmission was accurately reported, under-reporting of minor bleeding events is a possibility. Although we included late bleeding as any bleeding event within 30 days postdischarge, the definition of late bleeding was variable in the published reports. The clinical implication of minor bleeding after TAVR was assessed in this study; however, some investigations were not considered for such minor bleeding complications.

AF was a strong predictive factor of late bleeding after TAVR.^9^ About 10% of the patients had bleeding complications beyond 2 years after TAVR, but this percentage should not be overstated because the number of patients followed at a very late phase was limited. The antithrombotic regimen comprising SAPT/DAPT and OACs will need to be changed over time during the follow-up period. The antithrombotic therapy was retrospectively analyzed in detail in patients with late bleeding. However, complete data about the dynamic change of drug information were hard to collect. Therefore, we could not provide a definite conclusion concerning the drug regimen. The antithrombotic therapy at discharge could not be evaluated in the multivariate analysis. Because this point is beyond the scope of our study, a further randomized study focusing on antithrombotic therapy after TAVR is needed.

## Conclusions

The cumulative incidence of all and major late bleeding in the Asian cohort were 7.4% and 5.2% at 3 years after TAVR. Moreover, late bleeding events significantly increased long-term mortality. It is important to prevent bleeding complications, especially in the high-risk cohort. Further studies are required to establish the optimal medical therapy and management for improving individual patient care after TAVR.

Perspectives **COMPETENCY IN MEDICAL KNOWLEDGE:** The cumulative incidence of all and major late bleeding after TAVR in the Asian cohort were similar to those of the Western cohort. In addition, the late bleeding events were significantly associated with an increased risk of mortality after TAVR.

**TRANSLATIONAL OUTLOOK:** Although it is challenging to reduce the risk of late bleeding after TAVR, further studies are required to establish the optimal management for improving individual patient care after TAVR.

## Funding Support and Author Disclosures

The OCEAN-TAVI registry is supported by Edwards Lifesciences, Medtronic, Boston Scientific, Abbott Medical, and Daiichi-Sankyo Company. Drs Yamamoto, Tada, Naganuma, Shirai, Mizutani, Tabata, Ueno, Watanabe, and Hayashida are clinical proctors for Edwards Lifesciences and Medtronic. Drs Koyama and Takagi are clinical proctors of Edwards Lifesciences. Dr Yashima is a clinical proctor for Medtronic. All other authors have reported that they have no relationships relevant to the contents of this paper to disclose.
