# Supplementary material for: Incidence, Timing, and Causes of Late Bleeding After TAVR in an Asian Cohort
Source: JACC Asia. 2022 Sep 6;2(5):622–32. doi: 10.1016/j.jacasi.2022.04.007 (PMC9660329; doi:10.1016/j.jacasi.2022.04.007)

**Supplemental Table 1. Univariate analysis for the association between late bleeding and clinical findings**

|  | Univariate analysis | | |
| --- | --- | --- | --- |
| Explanatory variables | OR | 95% CI | *P* value |
| Baseline characteristics |  |  |  |
| Age (per 1 year) | 1.01 | 0.98-1.04 | 0.59 |
| Male | 1.24 | 0.87-1.78 | 0.24 |
| BMI | 1.90 | 0.71-5.01 | 0.20 |
| High CFS (≥4) | 1.59 | 1.10-2.30 | 0.014 |
| NYHA class III/IV (for I/II) | 1.71 | 1.20-2.43 | 0.003 |
| Hypertension | 0.75 | 0.63-1.96 | 0.16 |
| Diabetes | 0.99 | 0.66-1.51 | 0.99 |
| Pre-existing AF | 1.33 | 0.89-1.96 | 0.16 |
| Coronary artery disease | 1.15 | 0.79-1.68 | 0.48 |
| Peripheral artery disease | 1.36 | 0.87-2.13 | 0.18 |
| Pulmonary disease | 1.43 | 0.99-2.08 | 0.060 |
| Liver disease | 2.74 | 1.38-5.45 | 0.004 |
| Active cancer | 2.09 | 1.15-3.82 | 0.016 |
| STS score | 1.01 | 0.97-1.03 | 0.99 |
| eGFR | 1.01 | 0.99-1.01 | 0.93 |
| Hemoglobin | 0.94 | 0.85-1.05 | 0.27 |
| Low platelet count (<14.9×10^4^/μL) | 2.09 | 1.48-2.96 | <0.001 |
| Procedural bleeding complications | 1.01 | 0.68-1.52 | 0.95 |

OR, odds ratio; other abbreviations as in Table 1.

**Supplemental Table 2. Cox regression univariate analysis for the association between baseline variables and clinical outcomes**

|  | Univariate analysis | | |
| --- | --- | --- | --- |
| Explanatory variables | OR | 95% CI | *P* value |
| Baseline characteristics |  |  |  |
| Age (per 1 year) | 1.01 | 0.99-1.03 | 0.16 |
| Male | 1.71 | 1.41-2.08 | <0.001 |
| BMI | 0.93 | 0.91-0.96 | <0.001 |
| CFS ≥4 | 1.53 | 1.24-1.87 | <0.001 |
| NYHA class III/IV (for I/II) | 1.63 | 1.34-1.98 | <0.001 |
| Hypertension | 0.91 | 0.73-1.14 | 0.43 |
| Diabetes | 1.26 | 1.01-1.56 | 0.040 |
| Pre-existing AF | 1.42 | 1.15-1.77 | 0.001 |
| Coronary artery disease | 1.40 | 1.13-1.75 | 0.002 |
| Peripheral artery disease | 1.61 | 1.28-2.04 | <0.001 |
| Pulmonary disease | 1.43 | 1.16-1.76 | 0.001 |
| Liver disease | 2.62 | 1.77-3.88 | <0.001 |
| Active cancer | 1.82 | 1.29-2.58 | 0.001 |
| STS score | 1.03 | 1.03-1.04 | <0.001 |
| eGFR | 0.98 | 0.98-0.99 | <0.001 |
| Low platelet count (<14.9×10^4^/μL) | 1.60 | 1.32-1.95 | <0.001 |
| Types of bleeding |  |  |  |
| All late bleeding (for no all late bleeding) | 6.42 | 4.91-8.38 | <0.001 |
| GI bleeding for (for no GI bleeding) | 4.68 | 3.19-6.88 | <0.001 |
| Hemorrhagic stroke (for no hemorrhagic stroke) | 9.91 | 6.58-14.9 | <0.001 |
| Bleeding severity (reference as no bleeding) |  |  |  |
| Minor bleeding | 2.82 | 1.54-5.14 | 0.001 |
| Major bleeding | 8.70 | 6.51-11.6 | <0.001 |

Abbreviations as in Table 1.

**Supplemental Figure 1: The sub-group analysis of bleeding and ischemic stroke after TAVR**

(A) In the OAC group, major bleeding was significantly higher than ischemic stroke. (B) In the no OAC group, major bleeding was significantly higher than ischemic stroke. (C) In the AF group, major bleeding was significantly higher than ischemic stroke. (D) In the no AF groups, major bleeding was significantly higher than ischemic stroke. All major bleeding complications were significantly higher than those of ischemic stroke events in patients regardless of OAC use and AF.


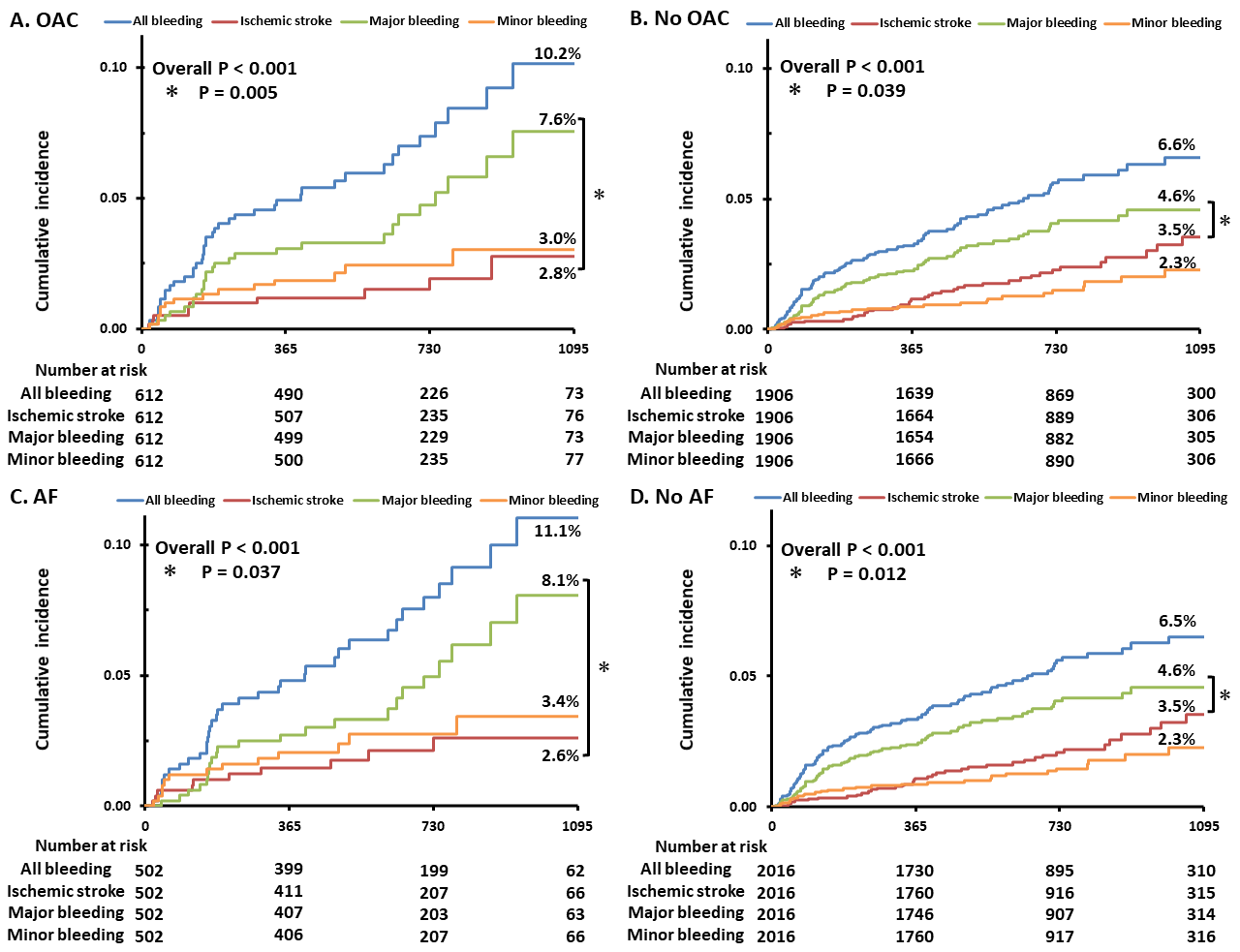

Supplement: Supplemental Figure 1 and Tables 1 and 2 [file mmc1.docx]
